# Psychodrama and Moviemaking in a Death Education Course to Work Through a Case of Suicide Among High School Students in Italy

**DOI:** 10.3389/fpsyg.2018.00441

**Published:** 2018-04-10

**Authors:** Ines Testoni, Lucia Ronconi, Lorenza Palazzo, Michele Galgani, Antonio Stizzi, Kate Kirk

**Affiliations:** ^1^Department of Philosophy, Sociology, Pedagogy and Applied Psychology (FISPPA), Università degli Studi di Padova, Padova, Italy; ^2^Hospice Aurelio Marena, Bitonto, Italy; ^3^Cork Counselling Services, Cork, Ireland

**Keywords:** death education, spirituality, psychodrama, movie making, alexithymia, representations of death, death anxiety

## Abstract

This study describes the psychological effects of an experience of death education (DE) used to explore a case of suicide in an Italian high school. DE activities included philosophical and religious perspectives of the relationships between death and the meaning of life, a visit to a local hospice, and psychodrama activities, which culminated in the production of short movies. The intervention involved 268 high school students (138 in the experimental group). Pre-test and post-test measures assessed ontological representations of death, death anxiety, alexithymia, and meaning in life. Results confirmed that, in the experimental group, death anxiety was significantly reduced as much as the representation of death as annihilation and alexithymia, while a sense of spirituality and the meaning of life were more enhanced, compared to the No DE group. These improvements in the positive meaning of life and the reduction of anxiety confirmed that it is possible to manage trauma and grief at school with death education interventions that include religious discussion, psychodrama and movie making activities.

## Introduction

As Gorer (1965) affirmed in the article “The Pornography of Death” that sex and death exchange positions of the “forbidden” and that the censorship of death issues is widely spread in contemporary Western civilization. Certainly Western post-modern societies are widely characterized by intercultural differences, and many expressions of religiosity run in parallel with atheism and agnosticism, so it is important to be cautious in affirming that death and dying are totally concealed. However, it is possible to state that in this complex scenario the general trend is toward a form of significant censorship of real mortality, arguing that this inevitably produces notable effects in individual's lives. Indeed, as cultural psychology shows, cultures shape the psychological processes of their members because the mind is shaped by beliefs, representations, traditions and social practices (Bruner, 1990; Shweder, 1991; Cole, 1996; Heine, 2011).

In the current post-modern and secularized culture, traditional and religious reflection on the afterlife has progressively eroded to the point that finding meaning in death and dying creates difficulties for people (Doka, 2007; Ronconi et al., 2009; Testoni, 2016). Indeed, whereas in the past the meaning of life processes were practiced informally in the families, it seems that nowadays it is necessary to plan specific educational activities to recover these profound conversations (Wass, 2004; Testoni et al., 2017b). In fact, while the media portray such issues by resorting to sensationalist unrealistic events focusing on the exceptional, cruel and aggressive factors (Gilbert and Murray, 2007; Noppe, 2007; Sofka, 2007), parents find themselves unable to explain these life and death messages with their children because they are uncertain as to how to, and whether to, do so cautious about their child's ability and their competence (Butler, 2004; Fonseca and Testoni, 2011).

Secularized society has a reduced faith in an afterlife, which is one of the important remedies to death anxiety and this leads to some negative effects. In fact, as Terror Management Theory (TMT) demonstrates, mortality salience intensifies anxiety, and activates psychological defenses that censor the same triggers of angst (Greenberg et al., 1994; Solomon et al., 2000; Greenberg and Kosloff, 2008). From a TMT perspective, which is one of the important theories in death studies, all cultures develop beliefs that are shared by individuals and aimed at providing a sense that life is meaningful and offering an account of the origin of the universe, prescriptions for appropriate behavior, and assurance of immortality. A belief in immortality permits people to manage death anxiety in everyday life and assuming socially shared values keep the unconscious conflict at bay (Solomon et al., 2017).

Two forms of immortality are commonly shared: literal immortality as afforded by souls, heavens, afterlives, and reincarnations associated with all major religions; while symbolic immortality is achieved by being part of a human community and its history.

A recourse to the removal of mortality from awareness can lead to an unconscious suppression of thoughts and negative emotions, and this censorship could diminish the ability to cope with existential anxiety, developing an “alexithymia.” Our “black hole hypothesis” suggests that the tendency toward impoverished emotions (Bagby et al., 1994) or alexithymia could be related to a systematic cultural inhibition of dialogue on death and dying. In particular, young people are more vulnerable to the lack of competent reflections aimed at managing mortality salience; silence around the experience of suicide could be a major factor which exacerbates such an effect, because it gives the idea that suicide is insignificant Testoni et al., 2016.

In a broad sense, it can be said that formal death education responds to this need by promoting a reflection on existential themes and exploring contemporary concerns about death and beliefs in an afterlife (Kastenbaum, 2000; Wass, 2004).

### Death education and suicide in adolescence

The unexpected deaths of young people are experiences that can impact upon younger adolescents; sometimes they experience the sudden and tragic deaths of their peers, usually from accidents, illness or suicide, and when these experiences occur they find themselves unable to manage them and become isolated in their grief (Cupit and Meyer, 2014; Testoni et al., 2016b; Cupit and Kuchta, 2017). At this critical age, unfortunately, the incidence of suicidal behaviors combines with other important indications of suffering: self-harm, addiction and challenging behaviors that endanger life (Gosney and Hawton, 2007; O'Connor et al., 2010; Haw and Hawton, 2011). Young people who attempt suicide or adopt self-harming behaviors are struggling in all psychological areas; they are characterized by perceiving reality as unmanageable and horribly nonsensical (Brent et al., 1999; Sinclair and Green, 2005; Haw and Hawton, 2008; Brent, 2017). The need to reduce the problem is widely recognized and the role of the social context and sharing with families and peers is generally agreed (i.e., Goldston et al., 2008; Schwartz-Lifshitz et al., 2012). In this area, prevention policies discuss the possibility of transforming the school into a psycho-educational space that can become a protective factor against suicidal risk or enable the expression of any kind of loss and grief. These prevention strategies propose to implement pivotal activities that focus on existential experiences and shared reflections in order to improve cultural values aimed at the significance of life, health, and well-being (Daniel et al., 2006; Goldsmith et al., 2007; Stanley et al., 2009). These curricula ensure that teenagers have accurate information about death, the value of life that must be protected; they offer simultaneously the opportunity to express emotions appropriately and develop a balanced realistic representation of death, instead of a dreamlike phantasmagorical one (Edgar and Howard-Hamilton, 1994). Furthermore, the strategies permit the exploration of grief. Such interventions offer young people the opportunity to discuss, in the ordinary space of the classroom, everything that causes suffering, loss, anxieties, and fears, without creating trauma or further psychological problems (Alexander and Adlerstein, 1958; Kastenbaum, 1967; Moss, 2000). These experiences promote activities of storytelling aimed at sharing experiences to reduce social isolation (Fortune et al., 2008). At other times psycho-educational strategies are used for existential reflection, activities like narrative methods or art-therapy (Walsh, 1993; Manley and Leichner, 2003; Dezutter et al., 2009; O'Connor et al., 2009; Sun-Hyun and So-Jeong, 2014). In summary, the overall aims of these death education methods are: to provide information on death and a common and appropriate language in order to understand emotions; to create space to reflect on the meaning of life; and to strengthen rational and critical thinking abilities (Wass, 2004).

Psychodrama offers excellent techniques, useful both for the expression of grief and for death education. Initiated by Moreno, psychodrama is a method most commonly conducted in a group format (Orkibi, 2011; Cruz et al., 2016) it can be used in individual settings (Pio-Abreu and Villares-Oliveira, 2007). It involves the representation of subjective experiences using dramatic techniques, where the participants are encouraged to develop their spontaneity and creativity in order to solve, in a new way, a situation from which suffering arises (Moreno, 1953). In order to improve self-awareness, personal empowerment and positive relationships in young people psychodrama techniques have been used in school settings as well (Azoulay and Orkibi, 2015). Managing contemporary issues of death, dying and associated death anxiety is hampered by the lack of adequate dialogue on death and dying between mature grownups and developing adolescents.

## The present study

### The “black hole hypothesis” and aims of the death education project

Our main hypotheses are derived from the conviction that death education should be offered increasingly in high school curricula using psychodrama techniques. The main hypothesis of the present study was that adolescents who participated in death education experience would express their emotions better, with a higher sense of control over their death imagery and death anxiety (i.e., Kastenbaum, 2004; Currier et al., 2008). An important concern regarding death education is inherent in the fact that enhancement of mortality could intensify death anxiety. So we wanted to show that: (1) it is possible to realize death education experiences without producing negative effects, by exploring death thoughts with thoughts of immortality; (2) the relationship between spirituality/religiosity/depictions of death can reduce death anxiety, as proposed by TMT; (3) psychodramatic, verbal and artistic explorations of the fear of death can reduce alexithymia. In particular, we wanted to confirm the efficaciousness of immortality representation as a support in the managing death anxiety elicited by mortality salience.

## Method

### The activities

Subsequent to the conviction that death education is useful in opening a debate to explore the various ways of conceptualizing death and, hopefully, lead to fostering of greater understanding and clarification of personal value systems, a death education project was created in a Southern Italy village, where an adolescent student had committed suicide.

A death education project was created in a Southern Italy village, where an adolescent student had committed suicide; this was based on the conviction that death education is useful in creating a debate in which to explore the various ways of conceptualizing death, to lead to a greater understanding and clarification of personal value systems.

The project was presented as a way to encourage students to discuss death and associated spirituality; it also aimed to open dialog about and reflections on the afterlife. It was approved by the directors of the institutes, by teachers and by parents; in fact all the three high schools in the community decided to participate in the death education course. They were aware of both the aims of promoting a sense of life and exploring traumatic grief.

There were three psychologists who were expert in psychodrama and death education; two others had expertise in religious sciences and worked at a hospice in a nearby town and collaborated in the program. At the end of the classroom activities, each class visited the hospice and during that visit students could share their emotions, thoughts, convictions about the afterlife and existential reflections. Teachers, who had specific and related training, were involved as supporters in all the activities.

The activities of death education were divided into “formal” work (during the lessons in the classroom) and “informal” work (activities at school for production of pictures and short movies using teamwork). The formal work included lessons on death, on meditation (Western and Eastern traditions) paying particular attention to the protective messages from religions. The informal work included activities of looking at a film on meditation before dying, then creating a psychodrama that reproduced some aspects of the movie. The material was widely shared by students, teachers and psychologists and the narratives were fundamentally aimed at explicitly expressing all the emotions that derived from awareness of mortality. In order to assume in the “first person” and the existential condition of dying or living with the awareness of being mortal, at this stage, they engaged with the psychodrama techniques of doubling, soliloquy and role reversal.

At the next stage, the students were entrusted with the following tasks: (a) to form work groups and search for useful documents that seemed better able to describe death, loss, and grief; (b) to share in the group their sensations, feelings, thoughts, reflections on death and afterlife and to explore and expand the ideas inspired by the film and their texts; (c) to develop and produce a movie outlining their psychodrama experience and the contents of the training course, this was intended to manifest their meaning making about death, life and afterlife; (d) to plan their presentation for a final exhibition at school and for a conference jointly organized by the hospice palliative team with the Municipality. Before and after all the encounters, the psychologists facilitated the student's warm up and their sharing moments in the classroom.

The study followed American Psychological Association Ethical Principles and Code of Conduct and the principles of the Declaration of Helsinki; furthermore it was approved by the Ethics Committee of University of Padua. Participants were informed about the study aims and procedures and they were assured that participation was voluntary. The confidentiality of their responses was guaranteed. Informed consent was obtained from all participants and their parents.

### Participants

There were 268 student participants (57% girls) from 10th to 11th year of three high schools located in southern Italy. Of these, 138 participants were assigned to the Death Education group and attended the course (DE group) and 130 were assigned to a group of participants who did not participate in the course (No DE group). In each institute, about two classes were involved with the experimental DE group and two classes to the No DE group. The differentiation between DE group and No DE group was not random, because we preferred to involve the classes whose teachers were particularly motivated to participate in the project. In fact, as the primary requirement of the study was focused on the positive outcome of the experience, we specifically chose the classes of the motivated teachers for the experimental group. The No DE group was comprised students of the same institutes and the same year of study, but they did not participate in the course. Participant's characteristics are presented in Table 1.

**Table 1:** Characteristics of students in the two study groups.

**Variables**	**Groups**	
	**DE (*N* = 138)**	**No DE (*N* = 130)**
Age^a^	17.1(0.6)	17.2(0.6)
Gender female	75(54%)	77(59%)
Gender male	63(46%)	53(41%)
Scientific high school	20(14%)	27(21%)
Humanities high school	48(35%)	58(45%)
Professional institute	70(51%)	45(35%)
God believer	118(85%)	101(78%)
God non-believer	20(15%)	29(22%)
Religious practicing	37(27%)	37(29%)
Religious non-practicing	101(73%)	71(%)

a *For this variable is reported Mean and (SD)*.

*DE, Death education*.

### Measures

A socio-demographic questionnaire collected background information, including age, gender, grade, school, and religious attitude. The four self-report instruments below are Italian versions of standardized measures.

#### The testoni death representation scale (TDRS)

The Testoni Death Representation Scale (TDRS) (Testoni et al., 2015) is a short 6-item 5-point Likert scale measuring the ontological representations of death either as annihilation (i.e., the end of everything) or as a passage (i.e., belief in an afterlife). Lower scores indicate that the individual represents death as a passage, whereas higher scores represent death as total annihilation. These constructs have been used in research examining attachment (Codato et al., 2011), hypnosis (Facco et al., 2017), emotional impact of nursing (Zamperini et al., 2015) grief, and palliative care (Testoni et al., 2016a, 2017a).

#### The toronto alexithymia scale (TAS)

The Toronto Alexithymia Scale (TAS) (Bagby et al., 1994) is a 20-item instrument, expressed in a 5 point Likert scale, commonly used to measure problems in emotional competence. It consists of three subscales: the Difficulty Describing Feelings subscale used to measure difficulty explaining emotions; the Difficulty Identifying Feeling subscale used to measure difficulty in identifying emotions; and the Externally-Oriented Thinking subscale used to measure the tendency of individuals to focus their attention externally. TAS has been used to examine emotional understanding by gender, age, culture, mental and physical illness, empathy, deviance, and parental relationships. The TAS is also suitable for use with an adolescent population (Parker et al., 2010).

#### The personal meaning profile (PMP)

The Personal Meaning Profile (PMP) (Wong and Fry, 1998) measures individual perception of personal meaning in one's own life. PMP is based on the human need for life meaning which is individually constructed, as a culture-based cognitive system, and influences the choice of activities, objectives, personal values, and fulfillment in life. However, when these essential human needs are ensured individuals are more likely to cope better with their problems and to live a more rewarding life. The questionnaire consists of 57 items on a 7-levels Likert scale (from 1 = not at all, to 7 = a great deal), in seven subscales identifying the following dimensions of life meaning: Achievement (16 items, e.g., “I pursue worthwhile objectives”); Relationship (9 items; e.g., “I am highly regarded by others”; “I am trusted by others”), Religion (9 items, e.g., “I believe that life has an ultimate purpose and meaning”; “I believe that human life is governed by moral laws”); Self-Transcendence (8 items: e.g., “I seek higher values-values that transcend self-interest”; “I attempt to leave behind a good and lasting legacy”); Self-Acceptance (6 items: e.g., “I have learned that setbacks and disappointments are an inevitable part of life”; “I am at peace with my past”); Intimacy (5 items: e.g., “I have someone to share intimate feelings with”); Fair Treatment or Perceived Justice (4 items: e.g., “Life has treated me fairly”). The PMP was translated into Italian and the original 7-factor model was confirmed with good reliability scores for each scale (Testoni et al., 2017c).

#### The death anxiety scale (DAS)

The Death Anxiety Scale (DAS) (Templer, 1970) is one of the most commonly used tools for assessing anxiety death. It is a scale that provides a type of True/False response and consists of 15 items. The score ranges from 0 to 15, and the higher the score is, the higher the degree of anxiety of death of the responding individual. The Italian validation of this tool, carried out by Saggino and Kline (1996), has shown that while it mainly evaluates general anxiety it also emphasized the multidimensional nature of death anxiety by identifying three factors: the fear of death and dying; the passage of time; the fear of pain and operations.

### Data analysis

We conducted our analyses in two steps. Firstly, we examined the impact of the death education course on the death representation as annihilation, alexithymia, personal meaning in life and death anxiety by conducting a multivariate analysis of covariance on all baseline total scores, with age and gender as covariates and by mixed-design repeated measure analysis of variance to test within and between subject differences. Secondly, we tested the interaction effect of pre-test assessment in death education course. We calculated change scores by subtracting the post-test score from the pre-test score for TDRS, TAS, DAS, which were expected to diminish over time. We performed a linear regression analysis with change scores as dependent variables including gender, age, group, and all pre-test scores as predictors and only significant interactions between pre-test scores and group selected by stepwise method.

## Results

### Death education impact

Age did not have significant effect on baseline total scores, gender did have a significant effect at multivariate-level, Wilk's lambda = 0.92, *F*_(4, 260)_ = 5.50, *p* < 0.001, but only on baseline Death Anxiety at univariate-level, *F*_(1, 263)_ = 130.97 *p* < 0.001 (with a higher score for female than for male, Ms = 9.14 and 7.76, respectively), and no intergroup differences were found for baseline total scores, Wilks' lambda = 0.99, *F*_(4, 260)_ = 0.45, *p* = 0.770.

As seen in Table 2, there was a significant interaction between time and group effect on Death Representation as Annihilation. A follow-up analysis, with Bonferroni adjustment for multiple comparisons, indicated that the mean scores of the DE group significantly decreased over time Mdiff = −0.98, SE = 0.37, *p* = 0.018, whereas the No DE group significantly increased over time, Mdiff = 1.48, SE = 0.38, *p* < 0.001. Similarly, a significant interaction was also found between time and group on Alexithymia Total score and Alexithymia factors except Difficulty Identifying Feeling, the mean scores of the DE group significantly decreased over time: Mdiff = −3.28, SE = 0.78, *p* < 0.001 for Alexithymia Total score, Mdiff = −1.95, SE = 0.45, *p* < 0.001 for Difficulty Describing Feelings and Mdiff = −0.97, SE = 0.38, *p* = 0.022 for Externally-Oriented Thinking; whereas the No DE group significantly increased over time for Alexithymia Total score and Externally-Oriented Thinking, Mdiff = 2.16, SE = 0.80, *p* = 0.014, and Mdiff = 1.43, SE = 0.39, *p* < 0.001, respectively, and did not change for Difficulty Describing Feelings.

**Table 2:** Descriptive statistics for study variables by time in the two groups.

**Variables**	**DE**		**No DE**		**F_time_ × _group_**	**η*_*p*_*^2^**
	**T1 M (*SD*)**	**T2 M (*SD*)**	**T1 M (*SD*)**	**T2 M (*SD*)**		
**TESTONI DEATH REPRESENTATION SCALE (TDRS)**						
Death representation as annihilation	16.98(4.91)	16.00(4.59)	16.96(5.55)	18.44(5.28)	21.18^***^	0.07
**TORONTO ALEXITHYMIA SCALE (TAS)**						
Difficulty describing feelings	20.16(6.19)	18.21(6.07)	19.01(6.36)	19.46(6.07)	14.19^***^	0.05
Difficulty identifying feeling	14.79(4.20)	14.46(3.96)	14.78(4.39)	15.08(4.74)	1.66 ns	–
Externally-oriented thinking	17.96(4.51)	16.99(4.38)	18.31(4.75)	19.74(4.95)	19.27^***^	0.07
Total score	52.92(10.13)	49.65(10.40)	52.12(11.04)	54.28(11.59)	23.82^***^	0.08
**PERSONAL MEANING PROFILE SCALE (PMPS)**						
Achievement	5.29(0.87)	5.40(0.75)	5.28(0.92)	5.04(1.00)	13.96^***^	0.05
Relationship	5.18(0.92)	5.31(0.78)	5.17(0.97)	5.08(1.00)	5.44^*^	
Religion	4.26(1.03)	4.27(1.05)	4.04(1.23)	4.00(1.19)	0.13 ns	–
Self-transcendence	4.78(0.82)	4.91(0.78)	4.62(1.00)	4.59(1.00)	2.66 ns	–
Self-acceptance	4.50(0.94)	4.69(0.86)	4.61(0.99)	4.54(1.09)	5.35^*^	0.02
Intimacy	4.85(1.21)	5.15(1.17)	5.03(1.25)	4.82(1.30)	12.74^***^	0.05
Fair treatment	4.83(1.01)	4.77(0.99)	4.95(1.05)	4.75(0.98)	1.27 ns	–
Total score	4.81(0.66)	4.93(0.59)	4.83(0.74)	4.69(0.77)	11.38^**^	0.04
**DEATH ANXIETY SCALE (DAS)**						
Death anxiety	8.70(2.71)	7.98(2.76)	8.38(2.81)	8.23(3.00)	13.90^***^	0.05

* *p < 0.05*;

** *p < 0.01*;

*** *p < 0.001. ns, not significant*.

A significant interaction was found between time and group on Personal Meaning Total score and Personal Meaning subscales except Religion, Self-Transcendence and Fair Treatment, the mean scores of the DE group significantly increased over time for Personal Meaning subscales Self-Acceptance and Intimacy, Mdiff = 0.19, SE = 0.08, *p* = 0.032, and Mdiff = 0.30, SE = 0.10, *p* = 0.006, respectively, and did not change for Personal Meaning Total score and the other Personal Meaning subscales; whereas the No DE group significantly decreased over time for Personal Meaning Total score and Personal Meaning subscale Achievement, Mdiff = −0.14, SE = 0.05, *p* = 0.020 and Mdiff = −0.24, SE = 0.07, *p* < 0.001, respectively, and did not change for the other Personal Meaning subscales. Finally, a significant interaction was found between time and group on Death Anxiety: that the mean scores of the DE group significantly decreased over time Mdiff = −0.98, SE = 0.37, *p* = 0.018, whereas the No DE group did not change.

### Interaction effect of pre-test assessment in death education course

Regression analysis with change scores of Death Representation as Annihilation as dependent variable showed no interaction between group and any score at the pre-test (Table 3). There was a better positive change on Death Representation as Annihilation for females (β = 0.14 *p* = 0.009), students with high score on Death Representation as Annihilation at the pre-test (β = 0.55 *p* < 0.001), students with high score on Personal Meaning subscale Religion at the pre-test (β = 0.17 *p* = 0.018), students with low score on Alexithymia factor Difficulty Identifying Feeling at the pre-test (β = −0.15 *p* = 0.022) and, of course, for DE group (β = 0.24 *p* < 0.001).

**Table 3:** Regression analysis with gender, age, group, and all pre-test assessments predicting change scores of TDRS, Alexithymia factors, and DAS.

**Predictors**	**Change scores**				
	**TDRS**	**TAS1**	**TAS2**	**TAS3**	**DAS**
Gender (female = 1, male = 0)	0.14^**^	0.05	−0.04	0.10	−0.09
Age	0.00	−0.08	0.00	0.03	0.06
Group (DE = 1, No DE = 0)	0.24^***^	0.20^***^	0.10	0.27^***^	0.21^***^
**VARIABLES AT THE PRE-TEST**					
Death representation as annihilation (TDRS)	0.55^***^	−0.07	−0.05	−0.10	−0.11
Difficulty describing feelings (TAS1)	0.04	0.53^***^	−0.14^*^	−0.06	−0.05
Difficulty identifying feeling (TAS2)	−0.15^*^	−0.11	0.56^***^	−0.07	0.09
Externally-oriented thinking (TAS3)	0.02	−0.02	−0.06	0.48^***^	−0.10
Achievement (PMPS1)	0.03	−0.09	0.03	0.02	−0.06
Relationship (PMPS2)	−0.12	0.08	0.08	0.15	0.05
Religion (PMPS3)	0.17^*^	−0.01	0.00	−0.02	−0.19^*^
Self-transcendence (PMPS4)	0.14	−0.11	−0.11	0.08	0.14
Self-acceptance (PMPS5)	−0.04	0.10	0.04	−0.03	0.01
Intimacy (PMPS6)	−0.10	−0.04	0.01	−0.14^*^	0.06
Fair treatment (PMPS7)	0.06	0.04	0.06	−0.04	−0.06
Death anxiety (DAS)	−0.01	−0.08	−0.03	−0.15	0.46^***^
**INTERACTIONS BETWEEN VARIABLES AT THE PRE-TEST AND GROUP**					
PMPS2 × group				−0.20^*^	
PMPS6 × group		0.17^*^			
DAS × group				0.15^*^	
Total R-square	0.36	0.30	0.26	0.35	0.26

*Standardized coefficients are presented*.

* *p < 0.05*;

** *p < 0.01*;

*** *p < 0.001*.

Regression analysis with change scores of Alexithymia factor Difficulty Describing Feelings as dependent variable showed a significant interaction between group and Personal Meaning subscale Intimacy at the pre-test. There was a better positive change on Alexithymia factor Difficulty Describing Feelings for students in DE group with high score on Intimacy at the pre-test (β = 0.17 *p* = 0.038), and also for students with high score on Alexithymia factor Difficulty Describing Feelings at the pre-test (β = 0.53 *p* < 0.001) and, of course, for DE group (β = 0.20 *p* < 0.001).

Regression analysis with change scores of Alexithymia factor Difficulty Identifying Feeling as dependent variable showed no interaction between group and any score at the pre-test. But there was a better positive change on Alexithymia factor Difficulty Identifying Feeling for students with high score on Alexithymia factor Difficulty Identifying Feeling at the pre-test (β = 0.56 *p* < 0.001) and for students with low score on Alexithymia factor Difficulty Describing Feelings (β = −0.14 *p* = 0.042).

Regression analysis with change scores of Alexithymia factor Externally-Oriented Thinking as dependent variable showed two significant interactions, between group and Personal Meaning subscale Relationship at the pre-test and between group and Death anxiety at the pre-test. There was a better positive change on Alexithymia factor Externally-Oriented Thinking for students in DE group with high score on Death Anxiety at the pre-test (β = 0.15 *p* = 0.046) and for students in DE group with low score on Personal Meaning subscale Relationship at the pre-test (β = −0.20 *p* = 0.007), and also for students with high score on Alexithymia factor Externally-Oriented Thinking at the pre-test (β = 0.48 *p* < 0.001), for students with low score on Personal Meaning subscale Intimacy at the pre-test (β = −0.14 *p* = 0.021), and, of course, for DE group (β = 0.27 *p* < 0.001).

Finally, regression analysis with change scores of Death anxiety as a dependent variable showed no interaction between group and any score at the pre-test. There was a better positive change on Death anxiety for students with high score on Death anxiety at the pre-test (β = 0.46 *p* < 0.001), students with low score on Personal Meaning subscale Religion at the pre-test (β = −0.19 *p* = 0.011), and, of course, for DE group (β = 0.21 *p* < 0.001). Supplementary Material is presented in Appendix 1.

## Discussion

The results confirm our hypotheses. The first result is that students, who participated in the death education, using psychodrama techniques and artistic production of movies activities, reported a significant decrease in their representation of death as annihilation, while in the No DE group it significantly increased over time. The second result is that our research also confirmed what TMT literature has widely empirically demonstrated, namely the role of symbolic and literal representation of immortality as an effective buffer against the paralyzing effect of being aware of mortality (Solomon et al., 2017). This outcome is supported by the amelioration of the scores inherent to death anxiety in the experimental group compared with the No DE group. Literature has already shown the relationship between death cognition and the processes of death acceptance, which reduces death anxiety (Wong et al., 2004). However, our research confirms what the early classical studies on death education illustrated (Leviton and Fretz, 1979). All such effects can be supported by the confirmation of the increase of personal meaning in their own life, in particular in the dimensions of “Self-Acceptance” and “Intimacy,” obtained from the Personal Meaning by the experimental groups, compared to the No DE group. The importance of the meaning of life was also confirmed by the final structural model we obtained. Indeed, the research enabled us to recognize that personal meaning of life moderated alexithymia with positive change over time in the students who participated to the death education course. In particular, the impact of death education course on alexithymia reduction is more relevant for: students with a high score on Personal Meaning subscale Intimacy; students with low score on Personal Meaning subscale Relationship and students with a high score on Death Anxiety. There is also a moderating effect on alexithymia in the experimental group's positive change over time on PMP. Since, in particular, the increasing impact of death education course on the PMP is most relevant for students with high score on Alexithymia factor Externally-Oriented Thinking, it is useful to underline that death education course could help individuals whose attention is mostly focused externally.

Finally, a brief look at the importance of meaning between the fear of death and death acceptance, related to trauma in adolescence. As seen in the literature, many relationships overlap the meaning of death with the meaning of life, where the two dimensions influence both fear of death and death acceptance (Tomer, 2012). van Bruggen et al. (2017) found correlations between death anxiety, intolerance of uncertainty, neuroticism, and distress; they showed that ability in meaning making reduced the effects of death anxiety. Floyd et al. (2005) examined the relationship between exposure to trauma and attitudes toward existential issues. Their participants were undergraduate students who answered questions on exposure to trauma, fear of death, overall distress, and meaning in life. Results illustrated that those with a history of trauma exposure had higher levels of overall distress, but there were no differences in death anxiety or meaning in life. The results suggest that the positive outcomes (less fear of death and increased meaning in life) associated with exposure to traumatic events may be relatively rare, especially with younger adults. If we consider the experience of the suicide of a peer as traumatic, it is important to help adolescents explore and explain what is happening when they encounter such an experience. Because it is the conspiracy of silence in this case that simply abandons them to their own solipsistic negation of grief and suffering. Since it is normal for adolescents to suffer from death anxiety, fear of death and existential meaningless (Routledge and Juhl, 2010; To and Chan, 2016), not necessarily because of a traumatic loss, we think that our experience of death education could be useful. It provided an environment conducive to understanding the meaning of self-transformation in life and death. These developments suggest that the denial and fear of death are a meaningless distraction in the development of adolescent consciousness.

## Conclusion

Despite the increase of mortality salience implicated by the issues inevitably intrinsic to death education courses, our results confirm that an effective set of activities aimed to reflect on death and enhance parallel meaning making processes on existential themes, is suitable for adolescents. This outcome is in line with literature (Chikako, 2004), which shows that in early and middle adolescence the meaning of death for life may change. Specifically, our outcomes indicated that in this general post-modern and secular culture with the concomitant crisis in religious faith progressively supports the development of a conviction, in adolescents, that no afterlife exists beyond death. If we consider the importance of conviction in immortality with respect to buffering the terror of death, as indicated by the TM researchers, it is possible to confirm that a course of death education, aimed to reflect on the afterlife contents, can be useful in the management of death anxiety. Psychodrama and artistic activities in this context seem to be particularly effective in reducing anxiety and helping students to face these issues. Furthermore the “black hole hypothesis” has been confirmed. The improvement in a range of capabilities enabling recognition of emotions in oneself and in others, arising in part from the experimental group's components, supported the possible relationship between the conspiracy of silence about death and alexithymia. This was seen differently in the No DE one in which it seemed that such abilities weakened. In this sense, death education not only helps to manage death anxiety and does not exacerbate it, but also it improves emotional aptitudes, starting from the exploration of existential anguish. The experience of psychodrama and meditation enabled them to look toward their internal world, without anxiety.

When they encounter any kind of loss, death education can improve adolescents' abilities to promote the transformation of the distress into a post-traumatic growth, namely their ability to change a more or less severe stressor into an existential gain (Gerrish et al., 2009).

## Study limitations

While our results are encouraging, some limitations should be considered. First, it is impossible to generalize the outcome because the two groups of participants were not randomized. This can be a problem of almost all experiences of death education at school, since the educational context requires that all the teaching processes are tightly controlled before the start of the activities. Only when death education is widely integrated would it be possible to select randomized samples. The second limit is related to the lack of long term follow-up. In further studies it would be good to have a post-test follow up after 1 year, which would enable verification, or not, of the stability of the outcomes. Furthermore, as indicated by Beshai and Naboulsi (2004), to expand the concept of death anxiety it is necessary to supplement empirical research with qualitatively collected texts. Seeing the words of people, who have similar numerical scores, may yet show qualitatively different fears of death, and vice versa. Total reliance on empirical scales may not disclose the full tension between the dual poles of “life-death” anxiety. Future studies could analyze such relationships.

## Author contributions

All authors listed have made a substantial, direct and intellectual contribution to the work, and approved it for publication.

### Conflict of interest statement

The authors declare that the research was conducted in the absence of any commercial or financial relationships that could be construed as a potential conflict of interest.

## References

[B1] Alexander I. E. Adlerstein A. M. (1958). Affective responses to the concept of death in a population of children and early adolescents. J. Genet. Psychol. 93, 167–177. 10.1080/00221325.1958.10532416 13587942

[B2] Azoulay B. Orkibi H. (2015). The four-phase CBN Psychodrama model: a manualized approach for practice and research. Arts Psychother. 42, 10–18. 10.1016/j.aip.2014.12.012

[B3] Bagby R. M. Parker J. D. Taylor G. J. (1994). The twenty-item toronto alexithymia scale-I. Item selection and cross-validation of the factor structure. J. Psychos. Res. 38, 23–32. 10.1016/0022-3999(94)90005-1 8126686

[B4] Beshai J. A. Naboulsi M. A. (2004). Existential perspectives on death anxiety. Psychol. Rep. 95, 507–513. 10.2466/pr0.95.2.507-513 15587217

[B5] Brent D. A. (2017). Firearms access and adolescent suicide. J. Am. Acad. Child Adolesc. Psychiatry 56:S331 10.1016/j.jaac.2017.07.689

[B6] Brent D. A. Baugher M. Bridge J. Chen T. Chiappetta L. (1999). Age- and sex-related risk factors for adolescent suicide. J. Am. Acad. Child Adolesc. Psychiatry 38, 1497–1505. 10.1097/00004583-199912000-00010 10596249

[B7] Bruner J. (1990). Acts of Meaning. Cambridge, MA: Harvard University Press.

[B8] Butler J. (2004). Precarious Life: The Powers of Mourning and Violence. London: Verso.

[B9] Chikako T. (2004). Changes in attitudes toward death in early and middle adolescence. Jap. J. Dev. Psychol. 15, 65–76.

[B10] Codato M. Shaver P. R. Testoni I. Ronconi L. (2011). Civic and moral disengagement, weak personal beliefs and unhappiness: a survey study of the “famiglia lunga” phenomenon in Italy. Test. Psychom. Methodol. Appl. Psychol. 18, 87–97. 10.4473/TPM20.2.6

[B11] Cole M. (1996). Cultural Psychology: A Once and Future Discipline. Cambridge, MA: Harvard University Press.

[B12] Cruz A. S. Sales C. M. D. Moita G. Alves P. G. (2016). Towards the development of Helpful Aspects of Morenian Psychodrama Content Analysis System (HAMPCAS). Z. Psychodr. Soziomet. 15, 57–67. 10.1007/s11620-015-0314-9

[B13] Cupit I. C. Meyer K. J. (2014). Accidents and Traumatic Loss: The Adolescent Experience, Helping Adolescents Cope with Loss. Washington, DC: Hospice Foundation of America.

[B14] Cupit I. N. Kuchta O. (2017). Death version 2016: how children and adolescents are learning and grieving in cyberspace, in Children, Adolescents and Death: Questions and Answers, eds Stevenson R. G. Cox G. R. (New York, NY: Routledge/Taylor & Francis Group), 25–36.

[B15] Currier J. M. Holland J. M. Neimeyer R. A. (2008). Making sense of loss: a content analysis of end-of-life practitioners' therapeutic approaches. OMEGA J. Death Dying 57, 121–141. 10.2190/OM.57.2.a 18680886

[B16] Daniel S. S. Walsh A. K. Goldston D. B. Arnold E. M. Reboussin B. A. Wood F. B. (2006). Suicidality, school dropout, and reading problems among adolescents. J. Learn. Disabil. 39, 507–514. 10.1177/00222194060390060301 17165618

[B17] Dezutter J. Luyckx K. Hutsebaut D. (2009). Are you afraid to die? Religion and death attitudes in an adolescent sample. J. Psychol. Theol. 37, 163–173. 10.1177/009164710903700302

[B18] Doka K. J. (2007). Historical and contemporary perspectives on dying, in Handbook of Thanatology, ed Balk D. (London: Routledge), 19–25.

[B19] Edgar L. V. Howard-Hamilton M. (1994). Noncrisis death education in the elementary school. Element. School Guid. Couns. 29, 38–46.

[B20] Facco E. Testoni I. Ronconi L. Casiglia E. Zanette G. Spiegel D. (2017). Psychological features of hypnotizability: a first step towards its empirical definition. Int. J. Clin. Exp. Hypn. 65, 98–119. 10.1080/00207144.2017.1246881 27935462

[B21] Floyd M. Coulon C. Yanez A. P. Lasota M. T. (2005). The existential effects of traumatic experiences: a survey of young adults. Death Stud. 29, 55–63. 10.1080/07481180490483463 15726743

[B22] Fonseca L. M. Testoni I. (2011). The emergence of thanatology and current practice in death education. OMEGA J. Death Dying 64, 157–169. 10.2190/OM.64.2.d 22375350

[B23] Fortune S. Sinclair J. Hawton K. (2008). Adolescent's views on preventing self-harm. Soc. Psychiatry Psychiatr. Epidemiol. 43, 96–104. 10.1007/s00127-007-0273-1 17994177

[B24] Gerrish N. Dyck M. J. Marsh A. (2009). Post-traumatic growth and bereavement. Mortality 14, 226–244. 10.1080/13576270903017032

[B25] Gilbert K. R. Murray C. I. (2007). The family, larger systems and death education, in Handbook of Thanatology, ed Balk D. (London: Routledge), 345–353.

[B26] Goldsmith S. K. Pellmar T. C. Kleinman A. M. Bunney W. E. (eds.). (2007). Reducing Suicide: A National Imperative. Washington, DC: National Academies Press. 25057611

[B27] Goldston D. B. Molock S. D. Whitbeck L. B. Murakami J. L. Zayas L. H. Nagayama Hall G. C. (2008). Cultural considerations in adolescent suicide prevention and psychosocial treatment. Am. Psychol. 63, 14–31. 10.1037/0003-066X.63.1.14 18193978 PMC2662358

[B28] Gorer G. (1965). The pornography of death, in Death, Grief, and Mourning, ed Gorer G. (Garden City: Doubleday), 192–199.

[B29] Gosney H. Hawton K. (2007). Inquest verdicts: youth suicides lost. Psychiat. Bull. 31, 203–205. 10.1192/pb.bp.105.007773

[B30] Greenberg J. Kosloff S. (2008). Terror management theory: implications for understanding prejudice, stereotyping, intergroup conflict, and political attitudes. Soc. Pers. Psychol. Compass 2, 1881–1894. 10.1111/j.1751-9004.2008.00144.x

[B31] Greenberg J. Pyszczynski T. Solomon S. Simon L. Breus M. (1994). Role of consciousness and accessibility of death-related thoughts in mortality salience effects. J. Pers. Soc. Psychol. 67, 627–637. 10.1037/0022-3514.67.4.627 7965609

[B32] Haw C. M. Hawton K. (2008). Life problems and deliberate self-harm: associations with gender, age, suicidal intent and psychiatric and personality disorder. J. Affect. Disord. 109, 139–148. 10.1016/j.jad.2007.12.224 18221789

[B33] Haw C. M. Hawton K. (2011). Problem drug use, drug misuse and deliberate self-harm: trends and patient characteristics, with a focus on young people, Oxford, 1993–2006. Soc. Psychiatry Psychiatr. Epidemiol. 46, 85–93. 10.1007/s00127-009-0170-x 19936579

[B34] Heine S. J. (2011). Cultural Psychology. New York, NY: Norton & Company.

[B35] Kastenbaum R. (1967). The child's understanding of death: how does it develop? in Explaining Death to Children, ed Grollman E. A. (Boston, MA: Allyn & Beacon), 89–108.

[B36] Kastenbaum R. (2000). The Psychology of Death 3rd Edn. New York, NY: Spring Publishing.

[B37] Kastenbaum R. (2004). Death, Society, and Human Experience 8th Edn. Boston, MA: Pearson.

[B38] Leviton D. Fretz B. R. (1979). Effects of death education on fear of death and attitudes towards death and life. OMEGA J. Death Dying 9, 267–277. 10.2190/431F-4169-JTVW-87MP

[B39] Manley R. S. Leichner P. (2003). Anguish and despair in adolescents with eating disorders: helping to manage suicidal ideation and impulses. Crisis 24, 32–36. 10.1027//0227-5910.24.1.32 12809151

[B40] Moreno J. L. (1953). Who Shall Survive? Foundations of Sociometry, Group Psychotherapy and Socio-Drama. Oxford: Beacon House.

[B41] Moss B. R. (2000). Death studies at university: new approaches to teaching and learning. Mortality 5, 205–214. 10.1080/713686007

[B42] Noppe I. C. (2007). Life Span issues and death education, in Handbook of Thanatology, ed Balk D. (London: Routledge), 337–343.

[B43] O'Connor R. C. Rasmussen S. Hawton K. (2009). Predicting deliberate self-harm in adolescents: a six month prospective study. Suicide Life Threat. Behav. 39, 364–375. 10.1521/suli.2009.39.4.364 19792978

[B44] O'Connor R. C. Rasmussen S. Hawton K. (2010). Predicting depression, anxiety and self-harm in adolescents: the role of perfectionism and acute life stress. Behav. Res. Ther. 48, 52–59. 10.1016/j.brat.2009.09.008 19818954

[B45] Orkibi H. (2011). Using intermodal psychodrama to personalize drama students' experience: two case illustrations. J. Aesthet. Educ. 45, 70–82. 10.5406/jaesteduc.45.2.0070

[B46] Parker J. D. A. Eastabrook J. M. Keefer K. V. Wood L. M. (2010). Can alexithymia be assessed in adolescents? Psychometric properties of the 20-item Toronto Alexithymia Scale in younger, middle, and older adolescents. Psychol. Assess. 22, 798–808. 10.1037/a0020256 20804260

[B47] Pio-Abreu J. L. Villares-Oliveira C. (2007). How does psychodrama work? in Psychodrama: Advances in Theory and Practice, eds Clark B. Burmeister J. Maciel M. (New York, NY: Taylor and Francis), 127–137.

[B48] Ronconi L. Testoni I. Zamperini A. (2009). Validation of the Italian version of the reasons for living inventory. Test. Psychom. Methodol. Appl. Psychol. 16, 151–159. 10.4473/TPM.16.3.4

[B49] Routledge C. Juhl J. (2010). When death thoughts lead to death fears: mortality salience increases death anxiety for individuals who lack meaning in life. Cogn. Emot. 24, 848–854. 10.1080/02699930902847144

[B50] Saggino A. Kline P. (1996). Item factor analysis of the Italian version of the Death Anxiety Scale. J. Clin. Psychol. 52, 329–333. 10.1002/(SICI)1097-4679(199605)52:3<329::AID-JCLP11>3.0.CO;2-K 8835696

[B51] Schwartz-Lifshitz M. Zalsman G. Giner L. Oquendo M. A. (2012). Can we really prevent suicide? Curr. Psychiatry Rep. 14, 624–633. 10.1007/s11920-012-0318-3 22996297 PMC3492539

[B52] Shweder R. (1991). Thinking Through Cultures. Cambridge, MA: Harvard University Press.

[B53] Sinclair J. Green J. (2005). Understanding resolution of deliberate self harm: qualitative interview study of patients' experiences. BMJ 330:1112. 10.1136/bmj.38441.503333.8F 15843425 PMC557888

[B54] Sofka C. J. (2007). Death education: ethical and legal issues, in Handbook of Thanatology, ed Balk D. (London: Routledge), 355–367.

[B55] Solomon S. Greenberg J. Pyszczynski T. (2000). Pride and Prejudice: fear of death and social behavior. Curr. Dir. Psychol. Sci. 9, 200–204. 10.1111/1467-8721.00094

[B56] Solomon S. Testoni I. Bianco S. (2017). Clash of civilizations? Terror management theory and the role of the ontological representations of death in contemporary global crisis. Test. Psychom. Methodol. Appl. Psychol. 24, 379–398. 10.4473/TPM24.3.5

[B57] Stanley B. Brown G. Brent D. Wells K. Poling K. Curry J. (2009). Cognitive Behavior Therapy for Suicide Prevention (CBT-SP): treatment model, feasibility and acceptability. J. Am. Acad. Child Adolesc. Psychiatry 48, 1005–1013. 10.1097/CHI.0b013e3181b5dbfe 19730273 PMC2888910

[B58] Sun-Hyun K. So-Jeong W. (2014). The effect of clinical art therapy programs for adolescent suicide prevention. J. Korean Soc. School Health 27, 100–108. 10.15434/kssh.2014.27.2.100

[B59] Templer D. I. (1970). The construction and validation of a death anxiety scale. J. Gen. Psychol. 82, 165–177. 10.1080/00221309.1970.9920634 4394812

[B60] Testoni I. (2016). Psicologia del lutto e del morire: dal lavoro clinico alla death education [The psychology of death and mourning: from clinical work to death education]. Psicoterapia Scienze Umane 50, 229–252. 10.3280/PU2016-002004

[B61] Testoni I. Ancona D. Ronconi L. (2015). The ontological representation of death: a scale to measure the idea of annihilation versus passage. OMEGA J. Death Dying 71, 60–81. 10.1177/0030222814568289 26152027

[B62] Testoni I. De Cataldo L. Ronconi L. Zamperini A. (2017a). Pet loss and representations of death, attachment, depression, and euthanasia. Anthrozoös 30, 135–148. 10.1080/08927936.2017.1270599

[B63] Testoni I. Falletti S. Visintin E. P. Ronconi L. Zamperini A. (2016a). Il volontariato nelle cure palliative: religiosità, rappresentazioni esplicite della morte e implicite di Dio tra deumanizzazione e burnout [Volunteering in palliative care: religiosity, explicit representations of death and implicit representations of God between dehumanization and burnout]. Psicol. Della Salute 27–42. 10.3280/PDS2016-002002

[B64] Testoni I. Ghellar T. Rodelli M. De Cataldo L. Zamperini A. (2017b). Representations of death among Italian vegetarians: an ethnographic research on environment, disgust and transcendence. Eur. J. Psychol. 13, 378–395. 10.5964/ejop.v13i3.1301 28904591 PMC5590526

[B65] Testoni I. Parise G. Visintin E. P. Zamperini A. Ronconi L. (2016b). Literary plastination: from body's objectification to the ontological representation of death, differences between sick-literature and tales by amateur writers. Test. Psychom. Methodol. Appl. Psychol. 23, 247–263. 10.4473/TPM23.2.8

[B66] Testoni I. Sansonetto G. Ronconi L. Rodelli M. Baracco G. Grassi L. (2017c). Meaning of life, representation of death, and their association with psychological distress. Palliati. Supp. Care. [Epub ahead of print]. 10.1017/S1478951517000669 28789719

[B67] To S. Chan W. C. (2016). Psychometric evaluation of the Chinese version of the Existential Anxiety Questionnaire in a sample of Chinese adolescents living in Hong Kong. Child Youth Care Forum 45, 487–503. 10.1007/s10566-016-9347-0

[B68] Tomer A. (2012). Meaning and death attitudes, in The Human Quest for Meaning: Theories, Research, and Applications, eds Wong P. P. Wong P. P. (New York, NY: Routledge/Taylor & Francis Group), 209–231.

[B69] van Bruggen V. Ten Klooster P. Westerhof G. Vos J. de Kleine E. Bohlmeijer E.. (2017). The Existential Concerns Questionnaire (ECQ)—development and initial validation of a new existential anxiety scale in a nonclinical and clinical sample. J. Clin. Psychol. 73, 1692–1703. 10.1002/jclp.22474 28369920

[B70] Walsh S. M. (1993). Future images: an art intervention with suicidal adolescents. Appl. Nurs. Res. 6, 111–118. 10.1016/S0897-1897(05)80171-5 8239639

[B71] Wass H. (2004). A perspective on the current state of death education. Death Stud. 28, 289–308. 10.1080/07481180490432315 15129687

[B72] Wong P. T. P. Fry P. S. (1998). The Human Quest for Meaning: A Handbook of Psychological Research and Clinical Applications. Mahwah, NJ: Lawrence Erlbaum.

[B73] Wong T. P. Reker G. T. Gesser G. (2004). Death attitude profile- revised: a multidimensional measure of attitudes toward death, in Death Anxiety Handbook: Research, Instrumentation, and Application, ed Neimeyer R. A. (Washington, DC: Taylor&Francis), 121–148.

[B74] Zamperini A. Paoloni C. Testoni I. (2015). The emotional labor of nursing: critical incidents and coping strategies [Il lavoro emozionale dell'assistenza infermieristica: Incidenti critici e strategie di coping]. Assistenza Infermieristica Ricerca 34, 142–148. 10.1702/2038.22142 26488930

