# Supplementary material for: Psychodrama and Moviemaking in a Death Education Course to Work Through a Case of Suicide Among High School Students in Italy
Source: Front Psychol. 2018 Apr 10;9:441. doi: 10.3389/fpsyg.2018.00441 (PMC5902682; doi:10.3389/fpsyg.2018.00441)
Supplement: Supplementary file 1 [file DataSheet1.docx]

Appendix 1

Supplementary material

Further results were followed, in particular inherent to the Personal Meaning Profile (see Table Appendix 1)

Regression analysis with change score of Personal Meaning subscale Achievement as a dependent variable showed a significant interaction between group and Personal Meaning subscale Relationship at the pre-test. There was a better positive change on Personal Meaning subscale Achievement for students in DE group with low score on Relationship at the pre-test (β = -.17 p = .023), and also for students with high score on Relationship at the pre-test (β = .20 p = .020), for students with low score on Achievement at the pre-test (β = -.50 p < .001) and, of course, for DE group (β = .23 p < .001).

Regression analysis with change score of Personal Meaning subscale Relationship as a dependent variable showed two significant interactions, between group and Alexithymia factor Externally-Oriented Thinking at the pre-test and between group and Personal Meaning subscale Relationship at the pre-test, the results indicated that there is a better positive change on Personal Meaning subscale Relationship for students in DE group with high score on Alexithymia factor Externally-Oriented Thinking at the pre-test (β = .16 p = .037) and for students in DE group with low score on Relationship at the pre-test (β = -.19 p = .013), and also for students with low score on Death Representation as Annihilation at the pre-test (β = -.17 p = .008), for students with low score on Alexithymia factor Difficulty Identifying Feeling at the pre-test (β = -.14 p = .036), for students with low score on Personal Meaning subscale Relationship at the pre-test (β = -.30 p = .001), and, of course, for DE group (β = .14 p = .010).

Regression analysis with change scores of Personal Meaning subscale Religion as a dependent variable showed a significant interaction between group and Personal Meaning subscale Self-Transcendence at the pre-test , the results indicated that there is a better positive change on Personal Meaning subscale Religion for students in DE group with low score on Self-Transcendence at the pre-test (β = -.19 p = .010), and also for females (β = .13 p = .035), students with low score on Religion at the pre-test (β = -.43 p < .001). Regression analysis with change over time on Personal Meaning subscale Self-Transcendence showed a significant interaction between group and Personal Meaning subscale Fair Treatment at the pre-test , the results indicated that there is a better positive change on Personal Meaning subscale Self-Transcendence for students in DE group with low score on Fair Treatment at the pre-test (β = -.20 p = .010), and also for students with high score on Relationship at the pre-test (β = .14 p = .035), for students with low score on Personal Meaning subscale Self-Transcendence at the pre-test (β = -.58 p < .001), and, of course, for DE group (β = .14 p = .011). Regression analysis with change over time on Personal Meaning subscale Self- Acceptance showed no interaction between group and any score at the pre-test , but results indicated that there is a better positive change on Personal Meaning subscale Self- Acceptance for students with high score on Personal Meaning subscale Fair Treatment at the pre-test (β = .13 p = .048), students with low score on Death Representation as Annihilation at the pre-test (β = -.14 p = .028), students with low score on Self- Acceptance at the pre-test (β =- .53 p < .001), and, of course, for DE group (β = .14 p = .012).

Regression analysis with change over time on Personal Meaning subscale Intimacy showed no interaction between group and any score at the pre-test, but results indicated that there is a better positive change on Personal Meaning subscale Intimacy for students with low score on Death Representation as Annihilation at the pre-test (β = -.17 p = .006), students with low score on Intimacy at the pre-test (β = - .47 p < .001), and, of course, for DE group (β = .19 p < .001). Regression analysis with change over time on Personal Meaning subscale Fair Treatment showed no interaction between group and any score at the pre-test, but results indicated that there is a better positive change on Personal Meaning subscale Fair Treatment only for students with low score on Fair Treatment at the pre-test (β = -.61 p < .001).

Table Appendix 1

Regression analysis with gender, age, group and all pre-test assessments predicting change scores of PMP scales

| Predictors | Change scores | | | | | | |
| --- | --- | --- | --- | --- | --- | --- | --- |
|  | PMPS1 | PMPS2 | PMPS3 | PMPS4 | PMPS5 | PMPS6 | PMPS7 |
| Gender (Female=1, Male=0) | .00 | .10 | .13* | -.01 | .08 | -.05 | -.01 |
| Age | .02 | .02 | .07 | .04 | -.10 | -.07 | -.05 |
| Group (DE=1, No DE=0) | .23*** | .14* | .08 | .14* | .14* | .19*** | .04 |
| *Variables at the pre-test* |  |  |  |  |  |  |  |
| Death Representation as Annihilation (TDRS) | -.11 | -.17** | -.09 | -.09 | -.14* | -.17** | -.11 |
| Difficulty Describing Feelings (TAS1) | -.02 | .04 | .01 | .04 | -.09 | -.03 | -.02 |
| Difficulty Identifying Feeling (TAS2) | -.09 | -.14* | -.13 | -.13 | -.09 | -.12 | -.08 |
| Externally-Oriented Thinking (TAS3) | -.04 | -.09 | .06 | .01 | -.06 | -.04 | -.03 |
| Achievement (PMPS1) | -.50*** | -.07 | .08 | .09 | .05 | -.11 | .05 |
| Relationship (PMPS2) | .20* | -.30** | -.11 | .14* | -.06 | .04 | .02 |
| Religion (PMPS3) | -.14 | -.06 | -.43*** | .02 | -.03 | .04 | -.01 |
| Self-Transcendence (PMPS4) | .10 | .10 | .16 | -.58*** | -.07 | -.12 | -.02 |
| Self- Acceptance (PMPS5) | .09 | -.03 | .03 | -.01 | -.53*** | .10 | .02 |
| Intimacy (PMPS6) | -.12 | -.08 | -.03 | -.02 | .01 | -.47*** | .09 |
| Fair Treatment (PMPS7) | -.08 | -.03 | .03 | .06 | .13* | .02 | -.61*** |
| Death Anxiety (DAS) | -.04 | -.02 | .03 | -.02 | -.08 | .02 | -.07 |
| *Interactions between pre-test scores and group* |  |  |  |  |  |  |  |
| TAS3 by group |  | .16* |  |  |  |  |  |
| PMPS2 by group | -.17* | -.19* |  |  |  |  |  |
| PMPS4 by group |  |  | -.19* |  |  |  |  |
| PMPS6 by group |  |  |  |  |  |  |  |
| PMPS7 by group |  |  |  | -.20* |  |  |  |
| DAS by group |  |  |  |  |  |  |  |
| *Total R-square* | *.31* | *.33* | *.23* | *.31* | *.31* | *.33* | *.33* |

*p<.05 **p<.01 ***p<.001
